# P-91. Fracture-Related Infections in South India: A Tertiary Care Center's Epidemiological, Clinical and Microbiological Profile

**DOI:** 10.1093/ofid/ofaf695.320

**Published:** 2026-01-11

**Authors:** Manju R Sebastian, Baker Fenn, A Anish, S Krishnakumar, Nirmal babu, Vasif Mayan, Netto George Mundadan, Athul Gurudas, Juby John

**Affiliations:** GOVERNMENT MEDICAL COLLEGE, KOTTAYAM, KOTTAYAM, Kerala, India; GOVERNMENT MEDICAL COLLEGE, KOTTAYAM, KOTTAYAM, Kerala, India; Government Medical College Kottayam, Kottayam, Kerala, India; GOVERNMENT MEDICAL COLLEGE, KOTTAYAM, KOTTAYAM, Kerala, India; Government Medical College Kottayam, Kottayam, Kerala, India; Government Medical College Kottayam, Kottayam, Kerala, India; Government Medical College, Kottayam, Kottayam, Kerala, India; GOVERNMENT MEDICAL COLLEGE, KOTTAYAM, KOTTAYAM, Kerala, India; Government Medical College Kottayam, Kottayam, Kerala, India

## Abstract

**Background:**

Fracture-related infections (FRIs) are increasingly common, with an estimated incidence of 1.8% and 27% for closed and open fractures, respectively. FRIs are challenging to treat due to their polymicrobial nature and prolonged duration of therapy. Defining local epidemiology will help guide empirical therapy especially in developing countries with high rates of resistant organisms.

**Figure:**
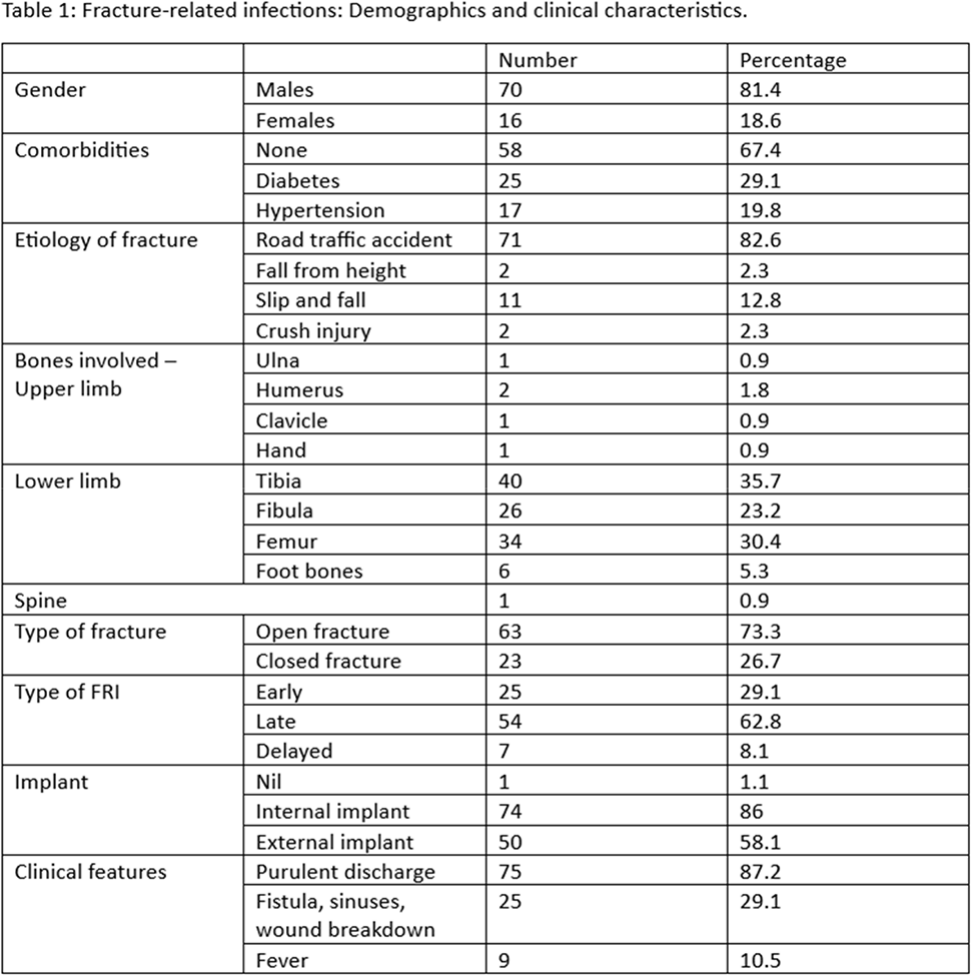

**Figure:**
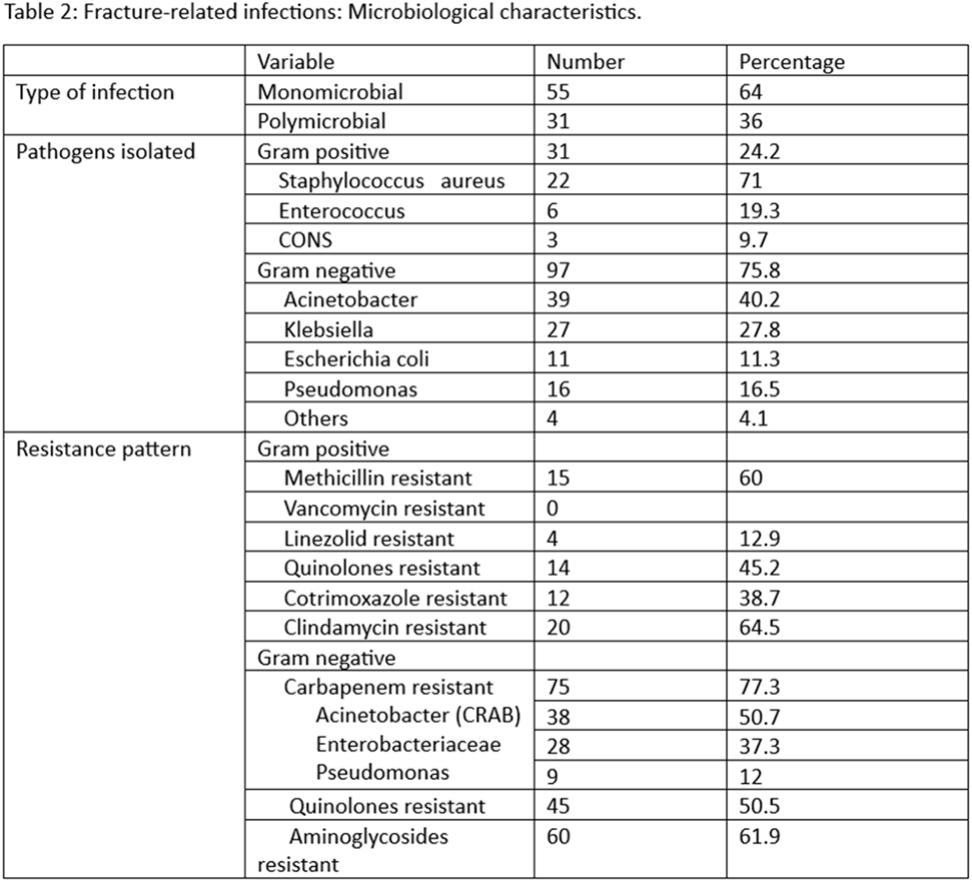

**Methods:**

The study was a single-centre, retrospective cohort study of patients diagnosed with FRI on presentation to the Orthopaedics department between 2024-2025. The FRI definition was based on recent consensus guidelines. Patients with prosthetic joint infections, involvement of sternum, skull or ribs and pathological fractures were excluded.

**Figure:**
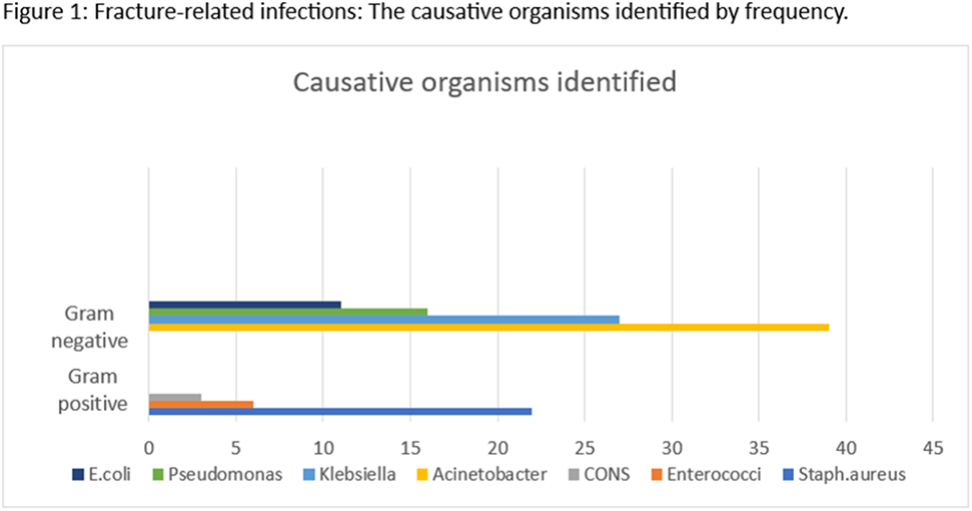

**Results:**

There were 86 patients included in the study and most were males (81%) with open fractures (73.3%). The most commonly involved bones were the tibia and femur (Table 1).

128 positive cultures were obtained from the patients and 75.8% were gram-negative and 24.2% were gram-positive organisms.

The gram-negative organisms isolated were *Acinetobacter baumanii* (40.2%), *Klebsiella pneumoniae* (27.8%), *Pseudomonas aeruginosa* (16.5%), and *Escherichia coli* (11.3%). The gram-positive organisms isolated were *Staphylococcus aureus* (71%), *Enterococcus species* (19.3%) and *Coagulase-negative Staphylococcus aureus* (9.7%) (Figure 1).

77.3% of the Gram-negative cases were resistant to carbapenems and *Acinetobacter baumanii* comprised 50.7%. No previous studies have shown carbapenem-resistant *Acinetobacter baumanii* as the predominant organism for FRIs which is likely due to under-reporting. As a tertiary referral center serving a rural population, prior antibiotic exposure in our patients likely contributes to the amplified selective pressure for antibiotic resistance.

On the other hand, none of the gram-positive organisms showed resistance to vancomycin (Table 2).

**Conclusion:**

There is an urgent need to address the rising carbapenem-resistance in developing countries. Empirical treatment strategies must adapt to provide adequate gram-negative coverage to improve patient outcomes. Further research should be directed to target the causes of this resistance and develop tailored antimicrobial stewardship and infection-control policies.

**Disclosures:**

All Authors: No reported disclosures

